# Roles for *Prlhr*/GPR10 and *Npffr2*/GPR74 in feeding responses to PrRP

**DOI:** 10.1016/j.molmet.2024.102093

**Published:** 2025-01-02

**Authors:** Yi Wang, Weiwei Qiu, Stace Kernodle, Carly Parker, Marc-Antonio Padilla, Jiaao Su, Abigail J. Tomlinson, Stephanie Oldham, Joss Field, Elise Bernard, David Hornigold, Christopher J. Rhodes, David P. Olson, Randy J. Seeley, Martin G. Myers

**Affiliations:** 1 Department of Internal Medicine, University of Michigan, Ann Arbor, MI, USA; 2 Department of Metabolism and Endocrinology, National Clinical Research Center for Metabolic Diseases, the Second Xiangya Hospital, Central South University, Changsha, 410000, China; 3 Department of Surgery, University of Michigan, Ann Arbor, MI, USA; 4 Early Cardiovascular Renal and Metabolism, BioPharmaceuticals, R&D, AstraZeneca, Cambridge, UK; 5 Hit Discovery, Discovery Sciences, BioPharmaceuticals R&D, AstraZeneca, Cambridge, UK; 6 Department of Pediatrics, University of Michigan, Ann Arbor, MI, USA; 7 Department of Molecular and Integrative Physiology, University of Michigan, Ann Arbor, MI, USA

**Keywords:** NTS, Food intake, Obesity, *Prlh*, *Prlhr*, *Npffr2*

## Abstract

**Objective:**

Several groups of neurons in the NTS suppress food intake, including *Prlh*-expressing neurons (NTS^Prlh^ cells). Not only does the artificial activation of NTS^Prlh^ cells decrease feeding, but also the expression of *Prlh* (which encodes the neuropeptide PrRP) and neurotransmission by NTS^Prlh^ neurons contributes to the restraint of food intake and body weight, especially in animals fed a high fat diet (HFD). We set out to determine roles for putative PrRP receptors in the response to NTS PrRP and exogenous PrRP-related peptides.

**Methods:**

We used animals lacking PrRP receptors GPR10 and/or GPR74 (encoded by *Prlhr* and *Npffr2*, respectively) to determine roles for each in the restraint of food intake and body weight by the increased expression of *Prlh* in NTS^Prlh^ neurons (NTS^PrlhOX^ mice) and in response to the anorectic PrRP analog, p52.

**Results:**

Although *Prlhr* played a crucial role in the restraint of food intake and body weight in HFD-fed control animals, the combined absence of *Prlhr* and *Npffr2* was required to abrogate the restraint of food intake in NTS^PrlhOX^ mice. p52 suppressed feeding independently of both receptors, however.

**Conclusions:**

Hence, each receptor can participate in the NTS^Prlh^-mediated suppression of food intake and body weight gain, while PrRP analog treatment can mediate its effects via distinct systems. While *Prlhr* plays a crucial role in the physiologic restraint of weight gain, the action of either receptor is capable of ameliorating obesity in response to enhanced NTS^Prlh^ signaling.

## Highlights

• *Prlhr* but not *Npffr2* restrains food intake and body weight in normal HFD-fed animals.
• Either *Prlhr* or *Npffr2* suffices to restrain obesity in response to increased NTS PrRP.
• PrRP analog can decrease feeding independently of *Prlhr* and *Npffr2*.
• There exists an additional pharmacologically relevant PrRP receptor.

## 1 Introduction

The current obesity pandemic represents an enormous challenge to human health and longevity worldwide [1]. Identifying new therapeutic targets to treat obesity will require understanding the brain systems that modulate feeding and contribute to body weight maintenance. While hypothalamic circuits contribute to the control of feeding and play important roles in maintaining long-term energy balance, recent findings reveal that brainstem circuits not only respond to gastrointestinal (GI) signals to mediate short-term feeding effects (e.g., meal termination), but also participate in the long-term physiologic control of food intake and body weight [[2], [3], [4], [5], [6], [7], [8]].

### 1.1 Dorsal vagal complex and NTS

Many of the most important food intake-controlling brainstem systems lie in the dorsal vagal complex (DVC), which includes the area postrema (AP), the *nucleus tractus solitarius* (NTS), and the dorsal motor nucleus of the vagus (DMV) [3,9]. The NTS receives direct input not only from the AP, but also from vagal sensory neurons that convey interoceptive signals from the gut, the airways, the vasculature, and other internal organs. The NTS processes these signals and relays them rostrally to control food intake and other behaviors. It also relays signals ventrally to the DMV (and elsewhere) to modulate GI physiology, cardiovascular parameters (e.g., heart rate and blood pressure), and other autonomic functions.

Several NTS cell types contribute to the suppression of feeding by gut peptide mimetics currently under development for weight loss therapy, including agonists for the calcitonin receptor (CALCR) and the related amylin receptor (AmyR- a complex of CALCR and a receptor activity modifying protein (RAMP)) [3,4,10,11]. Although some anorexigenic NTS neurons promote avoidance responses (suggesting that they mediate nausea-like aversive effects), *Lepr/Gcg*-expressing and *Calcr*-expressing NTS neurons (NTS^Calcr^ cells) suppress feeding without causing aversion-even when activated simultaneously-which completely abrogates feeding over 24 h [4,5,12]. Similarly, activating the subpopulation of NTS^Calcr^ neurons that contain prolactin releasing peptide (PrRP, encoded by *Prlh*; NTS^Prlh^ neurons) suppresses food intake without causing avoidance responses [2].

### 1.2 PrRP and its receptors

Injecting PrRP into the central nervous system (CNS) decreases feeding; conversely, disrupting *Prlh* in mice promotes obesity by increasing food intake and decreasing energy expenditure [[13], [14], [15], [16], [17], [18], [19]]. While *Prlh* in the dorsomedial hypothalamic nucleus (DMH, the other *Prlh*-expressing CNS site) promotes energy expenditure, *Prlh* in the NTS restrains food intake [15]. Furthermore, silencing NTS^Prlh^ neurons increases food intake and body weight, especially in mice fed a high fat diet (HFD) [2]. Consistently, overexpressing *Prlh* specifically in NTS^Prlh^ neurons (NTS^PrlhOX^ mice) abrogates the increased food intake and weight gain associated with HFD consumption and abrogates hyperphagia in genetic models of obesity [2,3]. Furthermore, the peripheral administration of long-acting analogs of PrRP suppresses food intake in obese rodents [19,20]. These findings suggest the potential usefulness of increased PrRP signaling generally and NTS^Prlh^ signaling specifically for the treatment of obesity. However, the central target for these interventions in the regulation of energy balance is still unknown.

Here, we reveal the specificity of NTS^Prlh^ neurons for the control of food intake-related parameters (as opposed to the modulation of locomotor activity). We also employ mice null for *Prlhr* and/or *Npffr2* (which encode the putative PrRP receptors GPR10 and GPR74, respectively) to define roles for each receptor in the control of food intake and body weight in NTS^PrlhOX^ mice and in PrRP analog responses.

## 2 Materials and methods

### 2.1 Animals

Mice were bred in our colony in the Unit for Laboratory Animal Medicine at the University of Michigan; these mice and the procedures performed were approved by the University of Michigan Committee on the Use and Care of Animals and in accordance with Association for the Assessment and Approval of Laboratory Animal Care and National Institutes of Health guidelines. Mice were provided with food and water *ad libitum* (except as noted below) in temperature-controlled rooms on a 12-hour light–dark cycle. For all studies, animals were processed in the order of their ear tag number, which was randomly assigned at the time of tailing (before genotyping).

*Prlh*^*Cre*^ mice (Jackson labs Strain #036747) and *Prlh*^*Flox*^ mice (Jackson labs Strain #036748) have been described previously [2]. *Gt(ROSA)26*^*Sortm5(CAG-Sun1/sfGFP)Nat/J*^ (Sun1-GFP reporter) mice for breeding were also from Jackson Labs (Strain #030952).

*Prlhr*^*KO*^, *Npffr2*^*KO*^, *Prlhr*^*Cre*^, and *Npffr2*^*Cre*^ mice were generated by the Molecular Genetics Core of the Michigan Diabetes Research Center. For the knockout alleles, the core designed and tested CAS9-dependent sgRNAs upstream and downstream of crucial sequences for each gene (the single coding exon of *Prlhr* and the first coding exon (exon 2) of *Npffr2*). For the cre alleles, the core designed and tested CAS9-dependent sgRNAs near the final codon of the relevant gene (at the end of the single coding exon of *Prlhr* and the final coding exon of *Npffr2*), and generated editing templates that replaced the stop codon of each with the sequences encoding a 2a peptide plus a cre with a nuclear localization sequence. CAS9 protein and the relevant sgRNAs (plus editing templates, for cre alleles) were injected into fertilized mouse embryos, and the fertilized embryos were implanted into pseudopregnant dams. Resultant pups were screened for deletion of the targeted sequences or the insertion of 2a-Cre sequences by PCR. For null alleles, sequences spanning the breakpoint were amplified and subjected to DNA sequencing. For cre alleles, sequences spanning from outside of the 5′ end to outside of the 3′ end of the editing template were amplified and sequenced. Positive animals were bred to C57Bl6/J mice and the resultant pups were rescreened and resequenced prior to propagation.

*Prlh*^*Cre*^ mice were bred onto the *Prlhr*^*KO*^ (10KO) and/or *Npffr2*^*KO*^ (74KO) backgrounds for experiments. Animals for study were generated by interbreeding *Prlh*^*Cre/Cre*^ mice on backgrounds homozygous for the appropriate receptor(s). *Prlhr*^*Cre*^ and *Npffr2*^*Cre*^ alleles were bred onto the *Gt(ROSA)*^*26Sortm2(CAG-mGCaMP3)Dbe*^ (Jackson Labs, Cat #030170) and *Rosa26*^*eGFP-L10a*^ [45] reporter alleles, respectively.

### 2.2 Viral reagents and stereotaxic injections

The previously described AAV-FLEX-Prlh vector [2] was produced by the University of Michigan viral vector core. AAV-FLEX-hM3Dq [46], AAV-GFP and AAV-CRE [47] were as previously described, and were also prepared by the University of Michigan viral vector core. The AAVs used in the manuscript were all serotype AAV8.

For AAV injection, following the induction of isoflurane anesthesia and placement in a stereotaxic frame, the skulls of adult mice were exposed. After the reference was determined, a guide cannula with a pipette injector was lowered into the injection coordinates (NTS: A/P −0.2; M/L ±0.2; D/V −0.2 from the obex) and 100 nL of virus was injected for each site using a picospritzer at a rate of 5–30 nL/min with pulses. Five minutes following injection, to allow for adequate dispersal and absorption of the virus, the injector was removed from the animal; the incision site was closed and sutured. The mice received prophylactic analgesics before and after surgery.

The mice injected with AAV-CRE or control viruses were allowed at least 1 week to recover from surgery before experimentation. The mice injected with AAV-FLEX-hM3Dq, and AAV-FLEX-Prlh or control viruses were allowed to recover for 3 weeks for virus expression before being subjected to additional interventions. *Prlh* reporter (*Prlh*^*Cre*^; *Gt(ROSA)26*^*Sortm5(CAG-Sun1/sfGFP)Nat/J*^) mice were injected with AAV-FLEX-Prlh or control virus to verify the restriction of PrRP overexpression to *Prlh* neurons. Because AAV-FLEX-Prlh virus was not tagged with a fluorophore, we sometimes co-injected it with a control GFP- or tdTomato-expressing AAV to aid in injection site validation. We examined PrRP-IR for all animals injected with control viruses or AAV-FLEX−Prlh. Mice with misplaced injections and/or that failed to display increased NTS PrRP-IR were discarded from analysis.

Other *Prlh*^*Cre*^ mice received intra-NTS injections of AAV^FLEX-hM3Dq^ (NTS^Prlh−Dq^ mice) to permit the artificial activation of NTS^Prlh^ neurons by the injection of clozapine-N-oxide (CNO).

### 2.3 Phenotypic studies

All mice were grouped and matched by age and body weight prior to surgery. For ablation studies, AAV-CRE injected *Prlh*^*Flox/Flox*^ mice and their controls were monitored weekly for food intake and body weight for 6 weeks post-surgery, after which they were transferred to a 60% HFD for an additional 6 weeks. For *Prlh* overexpression studies, *Prlh*^*Cre*^ mice injected with a control virus were designated as controls (Ctrl); while *Prlh*^*Cre*^ mice without gene knockouts were termed wild type (WT). The experiment included three parallel studies: *Prlh*^*Cre*^*; WT* or *Prlh*^*Cre*^; receptor knockout (KO) mice were injected with either a control virus (Ctrl-WT; Ctrl-KO) or AAV-FLEX-Prlh virus (PrlhOX-WT; PrlhOX-KO) and placed on HFD post-surgery. Food intake and body weight were monitored weekly. All measurements were taken at the same time point each week in both the ablation and overexpression studies.

For DREADD stimulation studies, NTS^Prlh−Dq^ mice and their controls, which were at least 3 weeks post-surgery, were rehoused on the first day and allowed a 3-day acclimatization period. Subsequently, all mice were treated with saline for 3 days, followed by 4 days of drug injections (CNO, 4936, Tocris, 1 mg/kg), and then returned to saline injections for an additional 3 days to allow for washout. All injections were administered twice daily, at the onset of the light and dark cycles, respectively. Food intake and body weight were monitored daily before the injections at the onset of the dark cycle.

In the short-term food intake response to agonist experiments, age- and body weight-matched mice from the same background were divided into parallel groups. All mice were fasted for 4 h prior to compound injection. Mice in each group were injected with either vehicle or agonists at the onset of the dark cycle, and food intake was monitored for 4 h post-injection. The injected volume was 10 times the body weight (10BW) for all mice.

### 2.4 Peptide synthesis

The long-acting PrRP analog p52 (Palm-SRTHR HS-Nle-EI RTPDI NPAWY ASRGI RPVGR Phe(pNO2)-amide) was synthesized on a Prelude (Gyros Protein Technologies) by automated synthesis using the Fmoc/^t^Bu protocol, purified by reversed-phase HPLC and characterized by single quadrupolar LC/MS using a Waters Mass Lynx 3100 platform. Analytical RP-HPLC spectra were recorded using an Agilent 1260 Infinity system. Peptide p52 was >99% pure after acetate exchange of the counterion.

### 2.5 Locomotor activity experiments

All mice were weighed before being placed in TSE metabolic cages. For NTS^Prlh−Dq^ mice and their controls, the mice were treated with saline after a 72-hour acclimatization period, followed by 1 day of drug injections (CNO, 4936, Tocris, 1 mg/kg). They were then returned to saline injections for an additional day to allow for washout. All injections were administered twice daily, at the onset of the light and dark cycles, respectively. For the p52 study, mice were treated with saline and p52 following the same protocol as the DREADD mice, but with injections administered once daily at the onset of the dark cycle. Locomotor activity was recorded by TSE PhenoMaster V7.7.9. and was calculated using Calrapp software: https://calrapp.org/cite.html.

### 2.6 RNA extraction and qPCR experiments

Fresh brain tissues were collected and flash-frozen in dry ice before being transferred to −80 °C until RNA extraction. The RNA extraction process was conducted strictly according to the RNeasy® Lipid Tissue Mini Handbook using the RNeasy® Lipid Tissue Mini Kit (Qiagen, 74804). RNA concentration was assessed using a NanoDrop 1000 (Thermo Scientific) prior to conversion to complementary DNA (cDNA). Approximately 1 μg of total RNA per sample was subjected to reverse transcription using the High-Capacity RNA-to-cDNA Kit (Thermo Fisher Scientific, Catalog #4387406). Quantitative PCR was performed using a Step OnePlus Real-Time PCR System (Applied Biosystems) in conjunction with TaqMan Gene Assays. The qPCR reactions were prepared with primers targeting the *Prlhr* or *Npffr2* genes plus a reference gene (*Gapdh*) for normalization. The thermal cycling conditions were optimized to ensure efficient amplification of cDNA, with fluorescence data collected at each cycle. The resulting cycle threshold (Ct) values were analyzed to determine relative gene expression levels, which were calculated using the 2^−ΔΔCt^ method. Primer Information: *Prlhr*: Mm01266991_s1 (FAM-MGB, Catalog #4331182, Thermo Fisher Scientific); *Gapdh*: Mm99999915-g1 (VIC-MGB, Catalog #4448490, Thermo Fisher Scientific); *Npffr2*: Mm01344955_m1 (FAM-MGB, Catalog #4351372, Thermo Fisher Scientific).

### 2.7 Perfusion and immunohistochemistry

Mice were anesthetized with a lethal dose of pentobarbital and transcardially perfused with phosphate-buffered saline (PBS) followed by 10% buffered formalin. Brains were removed, placed in 10% buffered formalin overnight, and dehydrated in 30% sucrose for at least 3 days. With use of a freezing microtome (Leica, Buffalo Grove, IL), brains were cut into 30 μm sections. Sections were treated with blocking solution (PBS with 0.1% triton, 3% normal donkey serum) for 1 h. The sections were incubated overnight at room temperature in primary antibody and exposed the next day with fluorescent secondary antibody to visualize proteins. Immunofluorescent staining was performed using primary antibodies (FOS, #2250, Cell Signaling Technology, 1:1000; GFP, GFP1020, Aves Laboratories, 1:1000; prolactin-releasing peptide (PrRP), H-008-52, Phoenix Pharmaceuticals, 1:500; dsRed, 632496, Takara, 1:1000), and species-specific Alexa Fluor-488, -568 or -647 conjugated secondary antibodies (Invitrogen, Thermo Fisher, 1:200). Images were collected on an Olympus (Center Valley, PA) BX53F microscope. Images were pseudocolored using Image J (NIH).

### 2.8 Quantification of PrPR-IR

For animals injected with AAV-GFP and AAV-CRE, the average number of PrRP-positive cell bodies was counted across three sections per animal. For NTS^PrlhOX^ mice and controls, acquired fluorescence images were converted to 8-bit grayscale format to standardize the intensity range for quantification. The NTS region was identified and manually outlined as the region of interest (ROI) using Image J (FIJI). The integrated density was then normalized to the ROI area to calculate the fluorescence intensity per unit area. Quantification was performed across three sections for each animal.

### 2.9 Statistics

Data are reported as mean ± standard error of the mean. Statistical analyses of physiologic data were performed with Prism software (version 10). Two-way ANOVA, One-way ANOVA, paired or multiple paired t-tests, unpaired t-test were used as indicated in the text and figure legends. *P* < 0.05 was considered statistically significant.

## 3 Results

### 3.1 Roles for NTS *Prlh*/PrRP signaling in feeding behavior and energy balance

**Figure 1:**
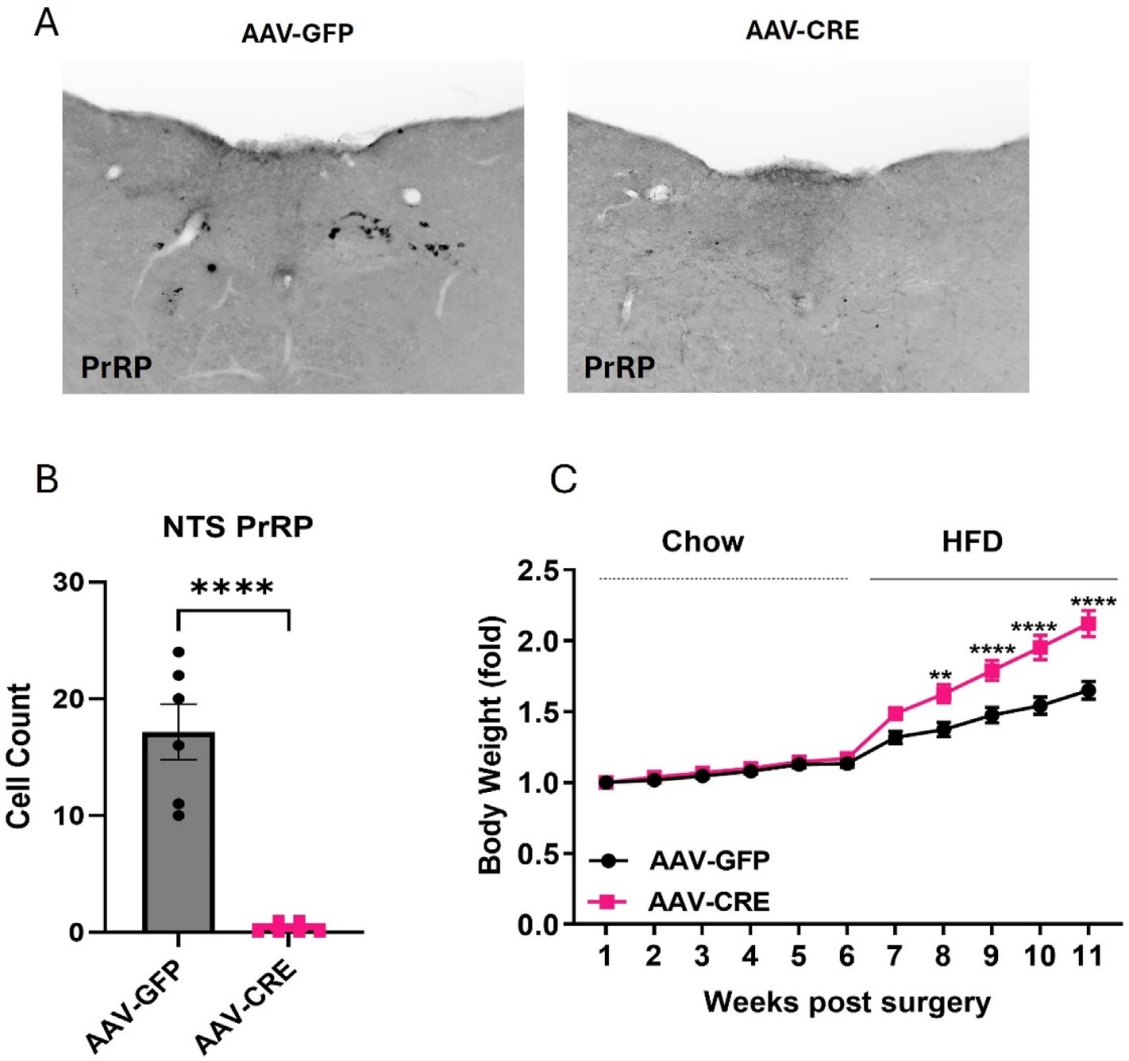
**Ablation of *Prlh* from the NTS promotes increased body weight in HFD-fed mice**. A. Representative image of PrRP-IR in NTS injection sites for AAV-GFP (left) and AAV-CRE (right) injected *Prlh*^Flox/Flox^ mice. B. Quantification of PrRP-IR cell bodies per section from AAV-GFP (n = 6) and AAV-CRE (n = 6) injected animals, as shown in (A). C. Body weight from AAV-GFP (n = 11; black line) and AAV-CRE (n = 8; red line) injected mice over 6 post-surgical weeks on chow and an additional 6 weeks on HFD. Body weight is normalized to the first week's measurement. All graphs: Shown is mean ± SEM. Two-way ANOVA, Sidak's multiple comparisons test and unpaired t-test were used; p values are shown for significant comparisons. ∗: *p* < 0.05, ∗∗: *p* < 0.01, ∗∗∗: *p* < 0.001, ∗∗∗∗: *p* < 0.0001. (For interpretation of the references to color in this figure legend, the reader is referred to the Web version of this article).

We previously showed that silencing NTS^Prlh^ neurons increased body weight gain in HFD-fed animals [2], establishing a physiologic role for these neurons in the restraint of body weight. To understand the potential role for endogenous *Prlh* (and its encoded peptide, PrRP) in this effect, we injected AAV-CRE into the NTS of *Prlh*^*Flox/Flox*^ mice to ablate of *Prlh* in the NTS (Figure 1). We found that the injection of AAV-CRE (but not control AAV-GFP) abrogated PrRP-immunoreactivity (-IR) in the NTS of *Prlh*^*Flox/Flox*^ mice (Figure 1A,B). While AAV-CRE-mediated ablation of NTS *Prlh* did not alter body weight compared to control AAV-GFP-injected mice under chow-fed conditions, AAV-Cre-injected mice gained substantially more weight than controls in HFD-fed animals (Figure 1C). Thus, endogenous NTS *Prlh*/PrRP restrains body weight accretion in HFD-fed animals.

**Figure 2:**
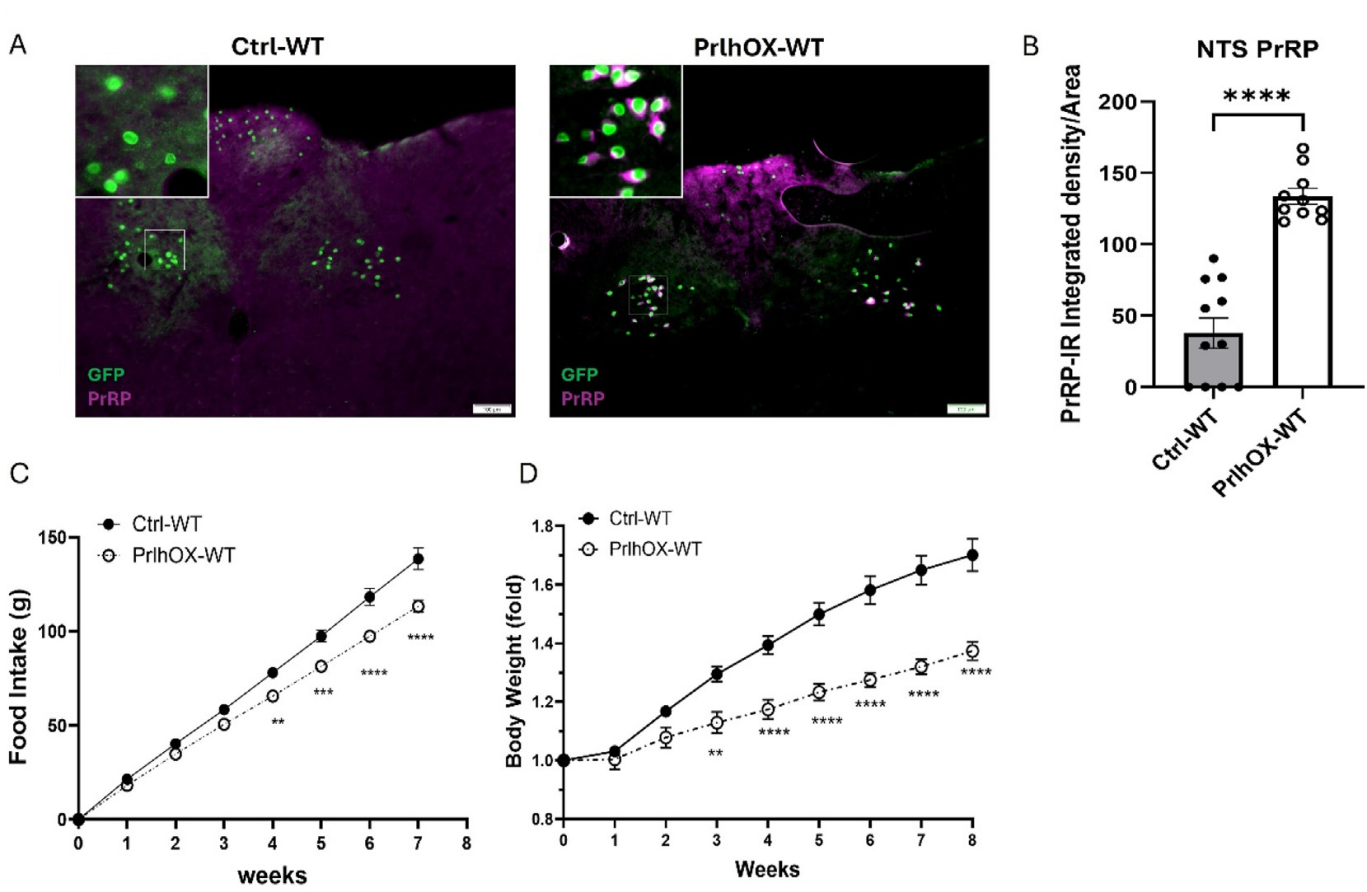
**Increased *Prlh* expression in NTS**^**Prlh**^**neurons restrains food intake and body weight gain in HFD-fed mice**. A. Representative image of PrRP-IR (magenta) and GFP (green) in the NTS of control (Ctrl-WT, left) or NTS^PrlhOX^ mice (PrlhOX-WT, right) on the Cre-dependent GFP-Sun1 reporter background. B. Quantification of PrRP-IR normalized per unit area of the NTS section from CTRL-WT (n = 11) and PrlhOX-WT (n = 10), as shown in (A). C, D Cumulative food intake (C) and normalized body weight (D) for HFD-fed control (Ctrl-WT, n = 16) and NTS^PrlhOX^ (PrlhOX-WT, n = 15) mice. Body weight is shown normalized to the first week's measurement. All graphs: Shown is mean ± SEM. Two-way ANOVA, Sidak's multiple comparisons test and unpaired t-test were used; All images were taken at same magnification; scale bar equals 100 μm. *P* values are shown for significant comparisons. ∗: *p* < 0.05, ∗∗: *p* < 0.01, ∗∗∗*p* < 0.001, ∗∗∗∗: *p* < 0.0001. (For interpretation of the references to color in this figure legend, the reader is referred to the Web version of this article).

To determine the ability of increased NTS *Prlh*/PrRP signaling to restrain food intake and body weight, we injected AAV-FLEX-Prlh [2] into the NTS of *Prlh*^*Cre*^ mice to amplify increase *Prlh* expression and PrRP content in NTS^Prlh^ neurons. We initially injected AAV-FLEX-Prlh into the NTS of *Prlh*^*Cre*^ mice on the *Gt(ROSA)26*^*Sortm5(CAG-Sun1/sfGFP)Nat/J*^ (cre-dependent GFP-Sun1) background to examine the distribution of increased PrRP-IR relative to GFP-labeled *Prlh* neurons in the NTS. In addition to identifying NTS Prlh neurons, this reporter line also revealed the existence of a few Prlh neurons in the AP, consistent with previous findings in rats [21]. This analysis also revealed approximately 4-fold increased PrRP-IR per unit area in the NTS of animals receiving AAV-FLEX-Prlh (NTS^PrlhOX^ mice) compared to controls and demonstrated that the increased PrRP-IR colocalized with *Prlh* neurons in the NTS (Figure 2A–B).

Because we have only observed long-term effects of NTS^Prlh^ neuron manipulations on food intake and body weight in HFD-fed (not chow-fed) animals (Figure 1 and [2]), the mice were recovered for one week on a normal chow diet and then switched to HFD for the determination of food intake (Figure 2C) and body weight gain (Fig. 2D) over the subsequent 8 weeks. NTS^PrlhOX^ mice exhibited decreased food intake and body weight gain compared to controls. Hence, not only does endogenous NTS *Prlh* restrain body weight during HFD-feeding, but enhanced *Prlh*/PrRP signaling by NTS^Prlh^ neurons can enhance this effect.

### 3.2 Generation of GPR10KO and GPR74KO mice

**Figure 3:**
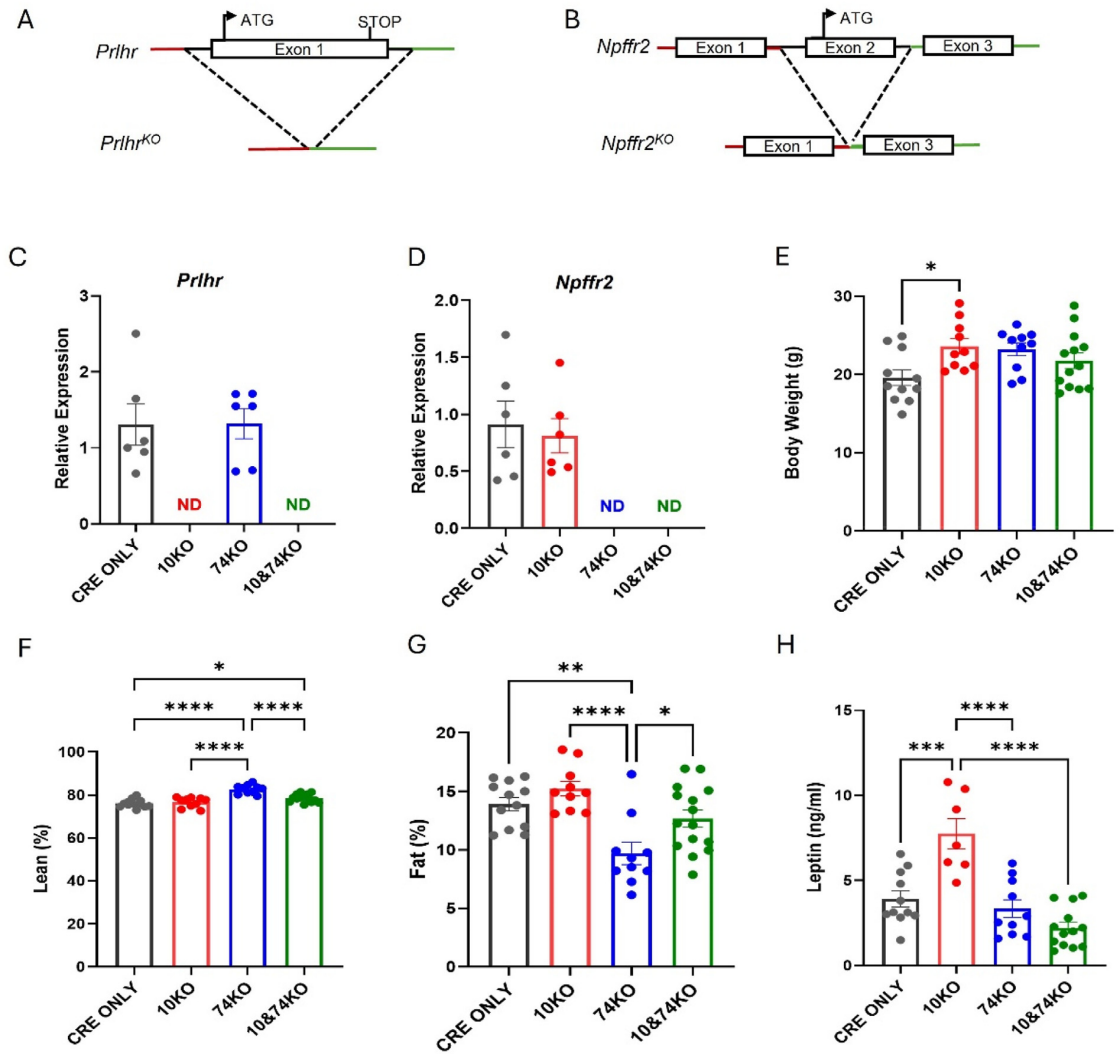
**Generation and baseline metabolic parameters in *Prlhr***^***KO***^**(10KO) and Npffr2KO (74KO) mice**. A, B Exon 1 of the *Prlhr* gene (A, encoding GPR10) and exon 2 of the *Npffr2* gene (B, encoding GPR74) were deleted in wild-type mice, respectively, using CRISPR technology. C, D Relative expression of *Prlhr* (C) and *Npffr2* (D) in the hypothalamus of wild-type mice and mice with individual and combined 10KO and 74KO genetic backgrounds. ND-not detected. E–H The body weight (E), lean mass percentage (F), fat percentage (G) and serum leptin (H) in 12-week-old chow fed mice on the individual or combined 10KO and 74 KO genetic backgrounds. All graphs: Shown is mean ± SEM. One-way ANOVA, Tukey's multiple comparisons test was used; p values are shown for significant comparisons. ∗: *p* < 0.05, ∗∗: *p* < 0.01, ∗∗∗*p* < 0.001, ∗∗∗∗: *p* < 0.0001.

Two CNS-expressed receptors for PrRP have been described- GPR10 (encoded by *Prlhr*) and GPR74 (encoded by *Npffr2*) [22,23]. To understand the roles for each of these in the reduced food intake and body weight exhibited by HFD-fed NTS^PrlhOX^ mice, we used CRISPR/Cas9-mediated gene editing to generate mice containing null alleles for *Prlhr* (*Prlhr*^*KO*^, Figure 3A) and *Npffr2* (*Npffr2*^*KO*^, Figure 3B). We bred these alleles, separately and together, onto the *Prlh*^*Cre*^ background for further studies.

While our manipulations of *Prlh*-expressing neurons target the NTS, NTS^Prlh^ neurons project strongly to rostral regions, including the hypothalamus, which contains many *Prlhr*-expressing and *Npffr2*-expressing neurons (e.g., Supplemental Fig. 1). To assess the potential ablation of *Prlhr* and *Npffr2* expression from these alleles, we thus dissected the hypothalamus from *Prlh*^*Cre/Cre*^;*Prlhr*^*KO/KO*^ (*Prlh*^*Cre*^;GPR10KO), *Prlh*^*Cre/Cre*^*;Npffr2*^*KO/KO*^ (*Prlh*^*Cre*^;GPR74KO), and compound *Prlh*^*Cre/Cre*^*;Prlhr*^*KO/KO*^*;Npffr2*^*KO/KO*^ (*Prlh*^*Cre*^;GPR10/74KO) mice, extracted RNA, and measured *Prlhr* (Figure 3C) and *Npffr2* (Figure 3D) mRNA expression by qRT-PCR. This analysis confirmed the lack of *Prlhr* mRNA expression in *Prlh*^*Cre*^;GPR10KO and *Prlh*^*Cre*^;GPR10/74KO mice and the lack of *Npffr2* mRNA expression in *Prlh*^*Cre*^;GPR74KO and *Prlh*^*Cre*^;GPR10/74KO mice, consistent with the genetic lesions in these animals.

We examined baseline metabolic parameters in 12-week-old control *Prlh*^*Cre*^, *Prlh*^*Cre*^;GPR10KO, *Prlh*^*Cre*^;GPR74KO, and *Prlh*^*Cre*^;GPR10/74KO mice (Figure 3E–H). In mice lacking GPR10, body weight was elevated compared to WT animals and leptin concentrations were significantly elevated compared to all other lines, consistent with the previously demonstrated role for GPR10 in physiologic energy balance [16,[23], [24], [25]]. In contrast, lean mass was increased and adiposity was decreased in *Prlh*^*Cre*^;GPR74KO mice compared to other strains (Figure 3F–G), consistent with multiple potential roles for GPR74 in energy balance, including previously-defined contributions to the restraint of lipolysis [26] and energy expenditure [27].

### 3.3 Roles for PrRP receptors in feeding and body weight responses in control and NTS^PrlhOX^ mice

**Figure 4:**
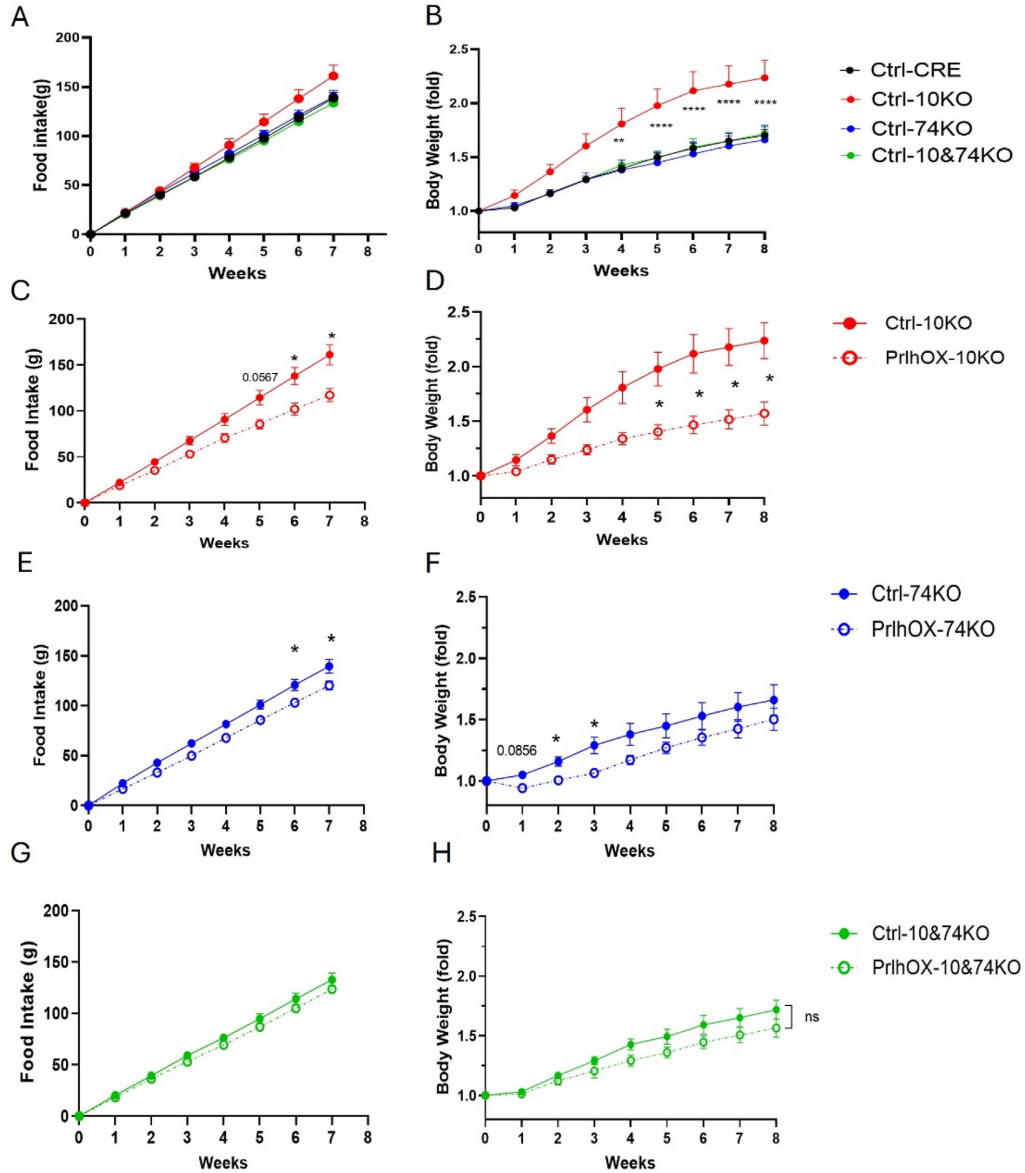
**Combined absence of GPR10 and GPR74 abrogates the suppression of food intake and body weight by NTS**^**PrlhOX**^**in HFD-fed mice**. (A–B) Cumulative food intake (A) and body weight (B) following surgery for Ctrl-CRE (n = 16), Ctrl-10KO (n = 6), Ctrl-74KO (n = 6), and Ctrl-10&74KO (n = 11) mice. (C–D) Cumulative food intake (C) and body weight (D) following surgery for control (Ctrl-10KO, n = 6; solid red line) and NTS^PrlhOX^ (PrlhOX-10KO, n = 6; dashed red line) mice on the GPR10 KO background. (E–F) Cumulative food intake (E) and body weight (F) following surgery for control (Ctrl-74KO, n = 6; solid blue line) and NTS^PrlhOX^ (PrlhOX-74KO, n = 6; dashed blue line) mice on the GPR74 KO background. (G–H) Cumulative food intake (left) and body weight (right) following surgery for control (Ctrl-10/74KO, n = 11; solid green line) and NTS^PrlhOX^ (PrlhOX-10/74KO, n = 12; dashed green line) mice on the combined 10KO/74KO background. Body weight is shown normalized to the first week's measurement for each strain. All graphs: Shown is mean ± SEM. Two-way ANOVA, Sidak's multiple comparisons test was used; *p* values are shown for significant comparisons. ∗*p* < 0.05, ∗∗: *p* < 0.01, ∗∗∗*p* < 0.001, ∗∗∗∗: *p* < 0.0001. (For interpretation of the references to color in this figure legend, the reader is referred to the Web version of this article).

We injected AAV-FLEX-Prlh or control AAV into the NTS of *Prlh*^*Cre*^ mice and *Prlh*^*Cre*^ mice on each of our receptor null lines (generating PrlhOX-WT, PrlhOX-10KO, PrlhOX-74KO, and PrlhOX-10/74KO mice and Ctrl-WT, Ctrl-10KO, Ctrl-74KO, and Ctrl-10/74KO mice, respectively) to study the effects of increasing PrRP in NTS^Prlh^ neurons in the absence of the PrRP receptors individually and collectively. Mice were recovered for one week after surgery and then switched to HFD; we measured food intake and body weight for these animals weekly for the subsequent 8 weeks (Figure 4).

We found that the effects of HFD on feeding and body weight were exacerbated in Ctrl-10KO mice compared to Ctrl-WT mice (Figure 4A,B), consistent with their baseline metabolic phenotypes (Figure 3E–G). Ctrl-10/74KO mice consumed similar amounts of food and gained similar amounts of weight on HFD compared to Ctrl-WT mice, suggesting that the lack of GPR74 essentially negated the effects of GPR10 ablation, resulting in a relatively normal metabolic phenotype.

Despite the crucial role for GPR10 in the physiologic restraint of food intake and body weight in control mice, *Prlh* overexpression in NTS^Prlh^ neurons dramatically reduced food intake and body weight in PrlhOX-10KO mice compared to Ctrl-10KO animals over the 8-week period of HFD feeding after surgery (Figure 4C,D). Thus, GPR10 is required for the normal control of food intake, energy expenditure, and body weight, but it is not required for the suppression of food intake by increased *Prlh*/PrRP in NTS^Prlh^ neurons.

Despite the decreased body weight and adiposity of GPR74-KO mice (Figure 4A,B), PrlhOX-74KO mice displayed decreased food intake and body weight relative to Ctrl-74KO animals (Figure 4E,F) and decreased food intake to levels similar to those observed PrlhOX-WT mice (Figure 4I). Although the body weight of PrlhOX-74KO mice was decreased compared to Ctrl-74KO control mice over the first 3 weeks of the study, this effect attenuated over the remainder of the 8-week study period (Figure 4F). This might reflect the previously described elevated energy expenditure and protection against fat storage in GPR74KO mice [26,28]. Hence, GPR74 is not required for the NTS^PrlhOX^-mediated suppression of food intake and body weight.

Interestingly, while neither GPR10 nor GPR74 alone was required for the suppression of food intake induced by increased PrRP in NTS^Prlh^ neurons, the combined absence of GPR74 and GPR10 abrogated the ability of NTS *Prlh* overexpression to decrease food intake and body weight in PrlhOX-10/74KO mice relative to Ctrl-10/74KO animals (Figure 4G,H). Thus, increased NTS *Prlh*/PrRP suppresses food intake and body weight via both GPR10 and GPR74.

### 3.4 Roles for PrRP receptors in feeding and body weight responses to p52

To determine roles for each PrRP receptor in the response to peripherally administered PrRP analogs, we synthesized p52-a variant of a previously reported PrRP analog [19] (p52 sequence Palm-SRTHR HS-Nle-EI RTPDI NPAWY ASRGI RPVGR Phe(pNO2)-amide) (Supplemental Figs. 2A and B) and confirmed its ability to activate each receptor in cultured cells (Supplemental Fig. 2C).

**Figure 5:**
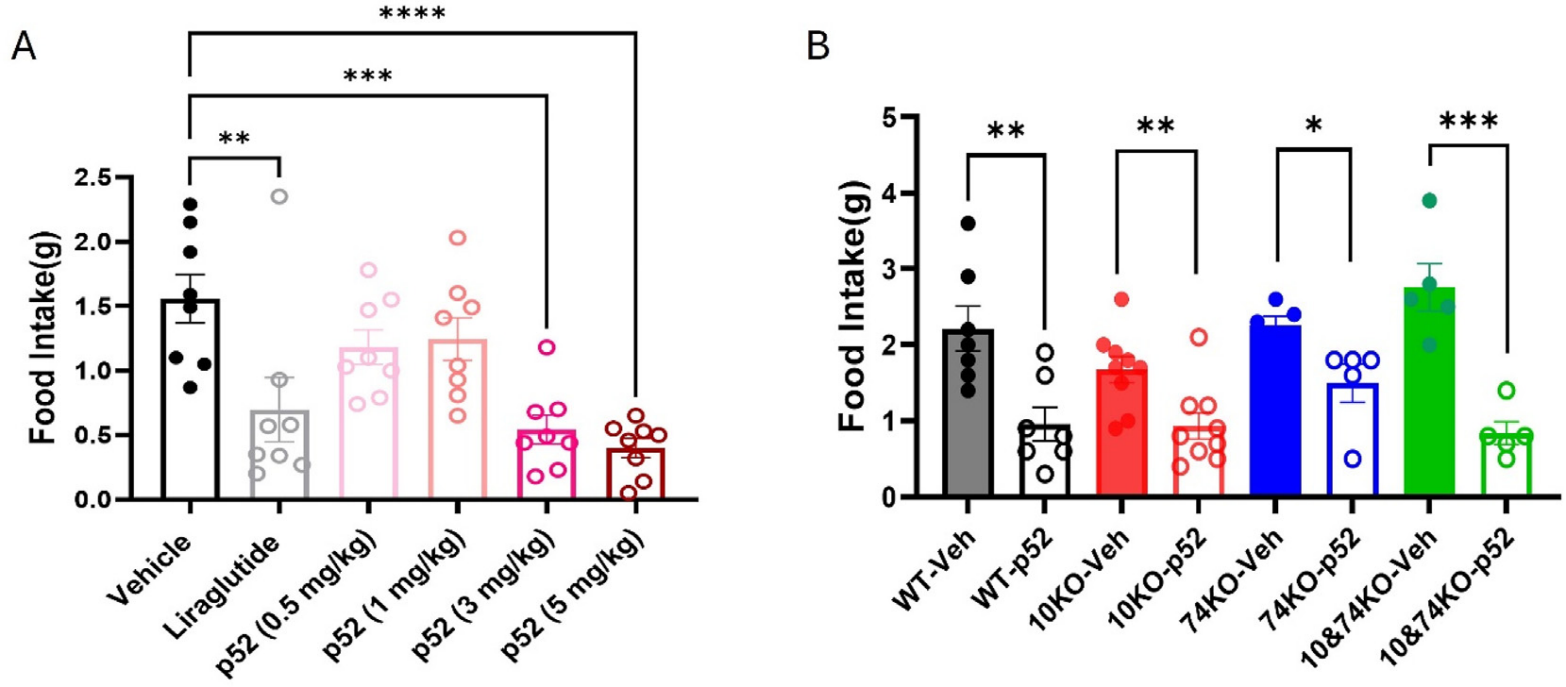
**p52 suppress food intake independently of GPR10 and GPR74 expression in mice**. A. Wild-type mice were treated with vehicle, liraglutide (dose) or the indicated concentration of p52 at the onset of the dark cycle and food intake was monitored over the subsequent 4 h (n = 8 per group). B Wild-type (WT) (n = 7), 10KO (n = 9), 74KO (n = 5), and 10/74KO (n = 5) mice were treated with vehicle or p52 (5 mg/kg) at the onset of the dark cycle and food intake was monitored over the subsequent 4 h. All graphs: Shown is mean ± SEM. One-way ANOVA, Dunnett's multiple comparisons test and unpaired t-test were used; *p* values are shown for significant comparisons. ∗: *p* < 0.05, ∗∗: *p* < 0.01, ∗∗∗*p* < 0.001, ∗∗∗∗: *p* < 0.0001.

We compared varying doses of p52 in their ability to decrease food intake over 4 h at the onset of the dark cycle, using the GLP1R agonist liraglutide as a positive control. This analysis revealed the robust reduction in food intake by p52 at doses of 3 mg/kg and 5 mg/kg (Figure 5A). Our pharmacokinetics analysis demonstrated that p52 remained detectable in the circulation for at least 4 h following a 5 mg/kg (but not 1.5 mg/kg) (Supplemental Fig. 2D). We thus tested the effect of p52 (5 mg/kg) on food intake in our single and compound PrRP receptor knockout mice (Figure 5B). Surprisingly, we found that p52 suppressed food intake in each of our PrRP receptor knockout lines, including 10/74KO mice. This finding suggests that p52 can inhibit food intake in mice via mechanisms independent of GPR10 and GPR74 and might mediate additional effects different than those mediated by NTS^Prlh^ neurons.

**Figure 6:**
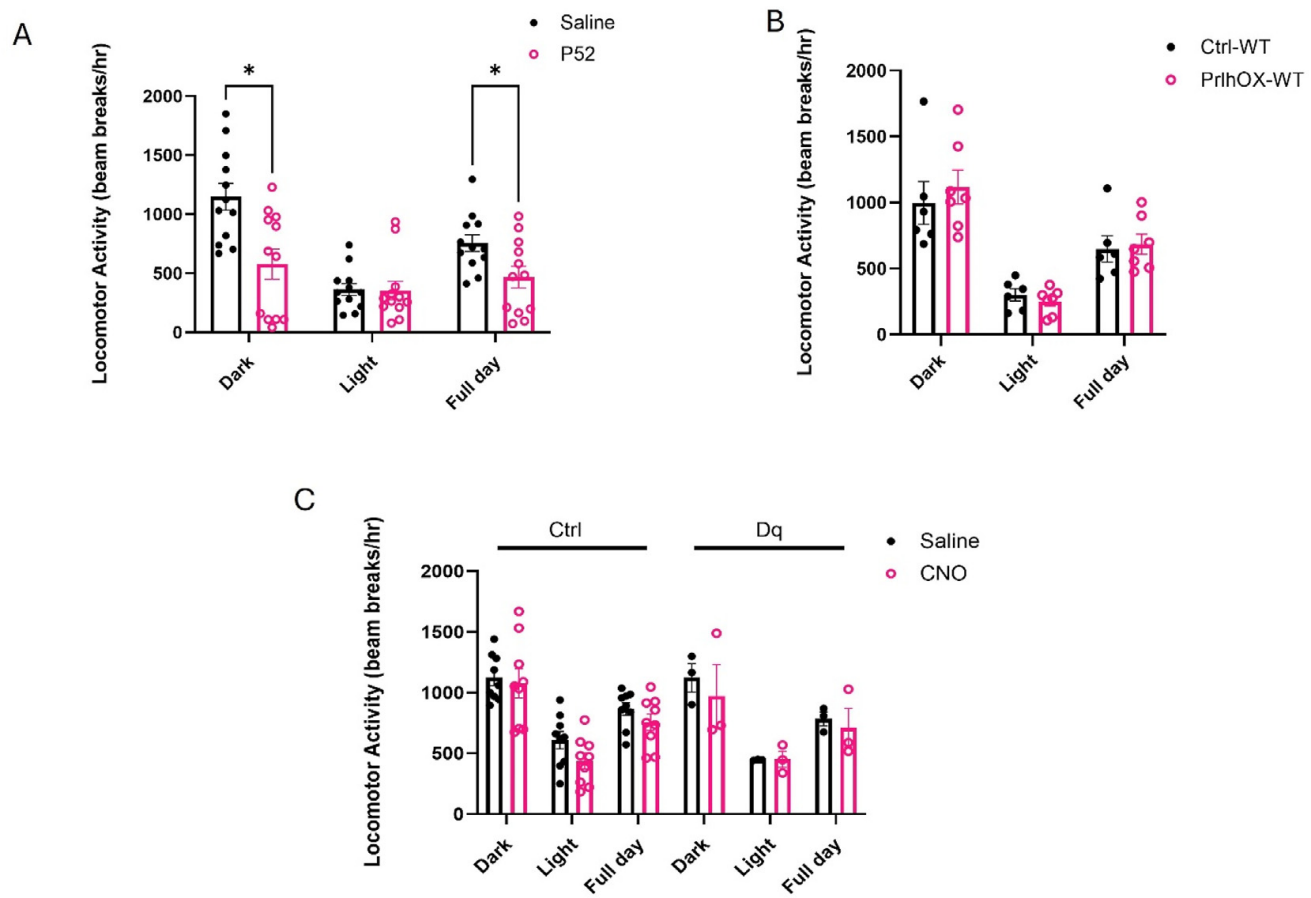
**p52, but not NTS**^**PrlhOX**^**or the activation of NTS *Prlh* neurons, suppresses locomotor activity in mice**. A. Locomotor activity in wild-type mice treated with vehicle and p52 (n = 12, 5 mg/kg IP), respectively. B. Locomotor activity in *Prlh*^*Cre*^ (n = 6; Ctrl-WT) and NTS^PrlhOX^ (n = 7, PrlhOX-WT) mice. C. Locomotor activity in control mice (Ctrl; n = 9) and NTS^Prlh−Dq^ mice (Dq; n = 3) treated with either saline or CNO (1 mg/kg IP) twice daily. All graphs: Shown is mean beam breaks per hour ± SEM for the light cycle, the dark cycle, and averaged over the entire 24-hour day. Multiple paired t-tests, Holm-Šídák method and Šídák-Bonferroni method were used; *p* values are shown for significant comparisons. ∗: *p* < 0.05, ∗∗: *p* < 0.01, ∗∗∗*p* < 0.001, ∗∗∗∗: *p* < 0.0001.

Because we noticed substantial behavioral changes, including decreased locomotor activity, in normal mice following the administration of p52, we examined the effects of p52 administration in metabolic cages capable of tracking locomotor activity. For comparison, we similarly examined locomotor activity in Ctrl-PrlhOX mice compared to controls, and in mice expressing the activating (hM3Dq) DREADD in NTS^Prlh^ neurons (NTS^Prlh−Dq^ animals) (Figure 6).

We found that p52 dramatically decreased locomotor activity in normal mice, primarily due to a decrease in activity during the dark (active) phase (Figure 6A). In contrast, PrlhOX-WT mice displayed no alterations in locomotor activity compared to Ctrl-WT mice (Figure 6B). Similarly, the administration of CNO to NTS^Prlh−Dq^ mice to activate NTS^Prlh^ neurons did not alter locomotor activity in the few mice that we tested (Figure 6C), despite producing the previously-reported dramatic suppression of food intake and body weight (Supplemental Fig. 3) [2]. Hence, while *Prlh*/PrRP from NTS^Prlh^ neurons suppresses food intake mainly via GPR10 and GRP74 and NTS^Prlh^ neurons appear to play roles specific to the control of ingestive behaviors, the PrRP analog p52 can suppress feeding via GRP10/74-independent mechanisms and promotes additional behavioral effects not observed in response to increased signaling by NTS^Prlh^ neurons.

## 4 Discussion

Our findings reveal that increasing *Prlh* expression/PrRP content in NTS^Prlh^ neurons can decrease food intake and body weight via either GRP10 or GPR74, although ablating both abrogates the food intake and body weight effects of increased NTS *Prlh*/PrRP signaling. Hence, pharmacologically targeting either receptor would be predicted to restrain feeding and body weight. In contrast, the PrRP analog, p52, suppress feeding independently of GPR10 and GPR74 and promotes behavioral effects not associated with increased signaling by NTS^Prlh^ neurons, suggesting that such peptides can restrain feeding (and promote additional effects) via other receptors.

### 4.1 PrRP receptor function

While the main focus of our study was to examine the requirement for GPR10 and GPR74 in the response to NTS PrRP and peripherally administered PrRP analog, we also observed baseline phenotypic differences in the knockout lines. Consistent with previous reports [24,29], the absence of GPR10 leads to increased body weight in chow-fed animals and this effect is exacerbated by HFD feeding, presumably due to their increased food intake. While we did not observe statistically significant differences in adiposity in chow fed GPR10KO mice compared to controls feeding, leptin concentrations were increased, consistent with increased adipose tissue mass.

Our GPR74KO mice exhibited similar food intake and body weight as wild-type mice (albeit with decreased adiposity). Similarly, others observed no difference or small decreases in body weight for chow-fed GPR74KO mice; as for our animals, others have observed decreased adiposity, as well-presumably due to increased energy expenditure [28,30]. Others have variably reported either increases or decreases in body weight and adiposity for these animals on HFD [28], [30]; while we observed a non-significant trend toward decreased body weight in HFD-fed GPR74KO mice. This variability of phenotype may result from differences in diet and/or housing conditions.

We found that the increased body weight caused by GPR10 deficiency was abolished in chow-fed GRP10/74KO mice; combined GPR10/74KO also normalized the increased food intake and body weight gain observed in GPR10KO animals during HFD-feeding. Hence, ablation of GPR74 appears to mitigate the hyperphagia of GPR10 mice. Others have observed some increased food intake in GPR10/74KO animals compared to wild types, however [31], suggesting that the effect may be incomplete and/or dependent upon the exact diet and housing condition under which the animals are assayed. It is also possible that the presumptive increase in energy expenditure in chow-fed GPR74KO [28,30] animals mitigates the decreased energy expenditure associated with the lack of GPR10 [26,27].

### 4.2 Targets for NTS PrRP

Given that ablation of GPR10 increases feeding while ablating GPR74 may produce small effects in the opposite direction, it makes intuitive sense that GPR10 would contribute to the restraint of feeding by NTS *Prlh* overexpression; the mechanisms underlying the role for GPR74 in the NTS *Prlh*-mediated restraint of feeding are less obvious. GPR74 is widely distributed and responds to NPFF (which is also widely expressed), as well as PrRP, however, and GPR74 mediates a host of effects [[32], [33], [34]]- some of which may increase food intake while others of which may decrease feeding. By overexpressing NTS *Prlh* specifically, we have presumably isolated a GPR74 circuit that restrains feeding.

The strongest projections from NTS *Prlh* neurons target the parabrachial nucleus (PBN), DMH, paraventricular thalamic nucleus (PVT), paraventricular hypothlaamic nucleus (PVH), and bed nucleus of the stria terminalis (BNST) [2]. To understand the potential neural targets for PrRP at these sites, we generated *Prlhr*^*Cre*^- and *Npffr2*^*Cre*^-reporter animals. Analyzing these animals revealed some *Prlhr*-expressing neurons in the PBN, DMH, and PVT, although the numbers were relatively small. We found relatively larger numbers of *Npffr2*-expressing neurons in the NTS, PBN, DMH, PVT, and BNST. These findings suggest that the GPR10-mediated effects of NTS *Prlh* are likely mediated by a few specialized neurons within the PBN, DMH, and/or PVT, while the GPR74-mediated effects might be more broadly distributed. Our examination of CNS FOS following IP p52 injection revealed no consistent FOS increases, unfortunately (data not shown), thus potential direct sites of p52 action in the CNS remain obscure. In the future, it will be important to identify roles for specific sites in the response to NTS-derived PrRP, as well as for PrRP-related peptides, such as p52.

### 4.3 Response to p52

While neither *Prlh* overexpression in NTS^Prlh^ neurons nor the pharmacologic activation of NTS^Prlh^ neurons altered locomotor activity, p52 decreased locomotor activity. Hence p52 mediates effects distinct from those mediated by NTS^Prlh^ neurons. Furthermore, in contrast to the requirement for the GPR10 and GRP74 in the response to overexpressing *Prlh* in NTS^Prlh^ neurons, the effects of p52 did not require these receptors-either alone or in combination. These findings suggest that peripherally dosed PrRP analogs like p52 can mediate their anorectic effects via neural pathways and receptors distinct from those used by NTS PrRP to decrease food intake.

While the overexpression of *Prlh* in NTS^Prlh^ neurons failed to significantly alter food intake or body weight in animals lacking both GPR74 and GPR10, we did observe non-significant trends toward decreases in these parameters in NTS^PrlhOX^;GPR10/74KO mice relative to Control GPR10/74KO mice. Hence, it remains possible that a receptor with lower affinity for PrRP than GPR10 and GPR74 (or that can mediate only smaller effects on food intake and body weight) could mediate a minor contribution to the NTS PrRP-mediated suppression of food intake and body weight.

PrRP is a member of the RFamide family of peptides and has measurable (albeit low) affinity for other RFamide peptide receptors, including GPR147 (also known as NPFFR1 or the RFRP-3/GnIH receptor) [33,34]. Furthermore, p52-related PrRP analogs exhibit substantial affinity for GPR147 [20]. Thus, PrRP analogs like p52 might act via GPR147 to decrease food intake. It is not clear whether p52 and other PrRP analogs can suppress food intake via GRP10 and/or GPR74 in the absence of GPR147, but it would be interesting to assess this possibility, as well as potential roles for each of these receptors in the locomotor suppression mediated by p52. Should GPR147 mediate this locomotor response, it would be useful to generate a GPR10 (and GPR74)-activating peptide without affinity for GPR147 for potential therapeutic use. Indeed, the known roles for GPR147 in the modulation of reproductive function and anxiety-like behaviors [35] caution against targeting this receptor for the therapy of obesity.

### 4.4 NTS^Prlh^ neuron function

Many feeding-related signals stimulate NTS^Prlh^ neurons: FOS studies [36,37] suggest the activation of these cells by CCK, feeding, and nutrient (e.g., leucine) treatment [[38], [39], [40], [41]]. A variety of stressors, including fear-provoking stimuli like restraint and foot shock, also increase FOS in these cells. Interestingly, *in vivo* monitoring of intracellular calcium (Ca^+2^_i_) flux suggests the activation of NTS^Prlh^ neurons by ongoing food ingestion during individual feeding bouts, rather than by the aggregate food consumed over many bouts [42]. Thus, physiologic NTS^Prlh^ neuron activity might limit the duration of feeding bouts in response to cues that dictate the need to acutely terminate feeding-such as nutrient ingestion and external threats. In the future it will be important to define roles for these cells in the cessation of feeding in response to such stressors.

While the activity of NTS^Prlh^ neurons can modulate feeding, NTS *Prlh* expression levels also impact the control of food intake. Furthermore, while glutamatergic transmission (not *Prlh* expression) mediates the suppression of food intake by the artificial activation of NTS^Prlh^ neurons, it is not required for long-term energy balance [2]. Rather, NTS *Prlh* expression (but not glutamatergic transmission by NTS^Prlh^ neurons) mediates the physiologic control of long-term food intake [2]. This may indicate that glutamatergic neurotransmission by NTS^Prlh^ neurons impacts mainly short-term parameters of feeding (e.g., meal duration and size), while the control of *Prlh* expression modulates satiation over longer periods of time and thus controls energy balance. Gonadal steroids, including estrogen and progesterone, increase NTS *Prlh* expression, as do stressors such as restraint [43,44]. Thus, the NTS expression of *Prlh* may contribute to the long-term control of body weight by these and other signals.

In addition to our need to definitively address the individual and overlapping roles played by NTS^Prlh^ neuron activity and *Prlh* expression for the physiologic short- and long-term control of food intake, it will also be important to define the downstream circuits and neural mechanisms by which NTS^Prlh^ neurons mediate these effects. NTS^Prlh^ neurons project most strongly to the lPBN, DMH, and PVT. Each of these NTS^Prlh^ neuron target regions participate in the control of feeding and contain *Prlhr*- and/or *Npffr2*-expressing cells. Going forward, it will be important to define the roles played by each of these regions in the responses to both NTS^Prlh^ neuron activity and PrRP signaling.

## Funding

Research support was provided by 10.13039/501100002822Central South University, NIH P01 DK117821 (MGM, DPO, and RJS), the Marilyn H. Vincent Foundation (MGM), and the 10.13039/100017243Michigan Diabetes Research Center, University of Michigan (NIH P30 DK020572, including the Molecular Genetics and Animal Studies Cores), the 10.13039/100018242Michigan Nutrition Obesity Research Center (MNORC, P30DK089503), and R01DK133140 (RJS).

## CRediT authorship contribution statement

**Yi Wang:** Writing – review & editing, Writing – original draft, Visualization, Methodology, Investigation, Formal analysis, Data curation, Conceptualization. **Weiwei Qiu:** Writing – review & editing, Investigation, Formal analysis, Data curation, Conceptualization. **Stace Kernodle:** Writing – review & editing, Investigation, Data curation. **Carly Parker:** Writing – review & editing, Investigation. **Marc-Antonio Padilla:** Writing – review & editing, Investigation. **Jiaao Su:** Investigation. **Abigail J. Tomlinson:** Project administration, Investigation, Data curation. **Stephanie Oldham:** Writing – review & editing, Resources, Methodology, Investigation. **Joss Field:** Writing – review & editing, Resources, Methodology, Investigation. **Elise Bernard:** Writing – review & editing, Resources, Methodology, Investigation. **David Hornigold:** Writing – review & editing, Resources, Methodology, Conceptualization. **Christopher J. Rhodes:** Writing – review & editing, Resources, Investigation, Conceptualization. **David P. Olson:** Writing – review & editing, Methodology, Conceptualization. **Randy J. Seeley:** Writing – review & editing, Resources, Project administration, Methodology. **Martin G. Myers:** Writing – review & editing, Writing – original draft, Supervision, Resources, Project administration, Funding acquisition, Data curation, Conceptualization.

## Declaration of competing interest

MGM and DPO receive research support from AstraZeneca, Eli Lilly, and Novo Nordisk. MGM has served as a paid consultant for Merck and received honoraria from Novo Nordisk. RJS has received research support from Novo Nordisk, Fractyl, Astra Zeneca, Congruence Therapeutics, Eli Lilly, Bullfrog AI, Glycsend Therapeutics and Amgen. RJS has served as a paid consultant for Novo Nordisk, Eli Lilly, CinRx, Fractyl, Structure Therapeutics, Crinetics and Congruence Therapeutics. RJS has received honoraria from AstraZeneca. RJS has equity in Calibrate, Rewind and Levator Therapeutics. SO, JF, EB, DH and CJR are employees of AstraZeneca and hold stock in the company. The authors declare that they have no other conflicts of interest.

## Data Availability

Data will be made available on request.
