# Supplementary material for: Roles for Prlhr/GPR10 and Npffr2/GPR74 in feeding responses to PrRP
Source: Mol Metab. 2025 Jan 2;92:102093. doi: 10.1016/j.molmet.2024.102093 (PMC11773474; doi:10.1016/j.molmet.2024.102093)
Supplement: Multimedia component 1 [file mmc1.pdf]

SUPPLEMENTAL FIGURES 1-3 FOR

Roles for *Prlhr*/GPR10 and *Npffr2*/GPR74 in Feeding Responses to PrRP

Yi Wang[1,2], Weiwei Qiu[1]*, Stace Kernodle[3], Carly Parker[1], Marc-Antonio Padilla[1], Jiaao Su[1], Abigail J. Tomlinson[1], Stephanie Oldham[4], Joss Field[4], Elise Bernard[5], David Hornigold[4], Christopher J. Rhodes[4], David P. Olson[1,6], Randy J. Seeley[3,7], and Martin G. Myers, Jr.[1,7]**

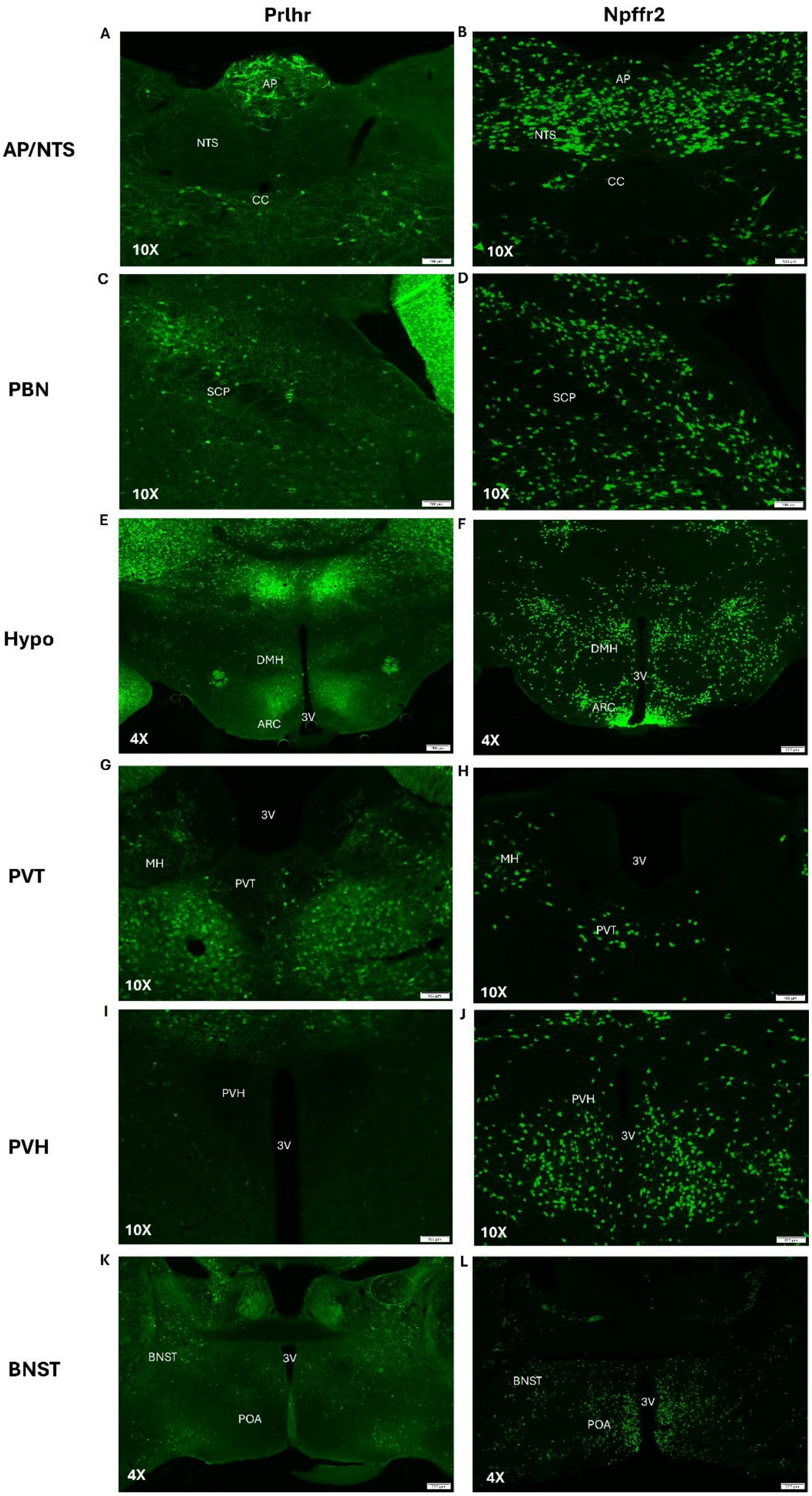

Prlhr
Npffr2
A
B
AP/NTS
AP
NTS
CC
10X
C
D
PBN
SCP
10X
E
F
Hypo
DMH
ARC
3V
4X
G
H
PVT
3V
MH
PVT
10X
I
J
PVH
PVH
3V
10X
K
L
BNST
BNST
3V
POA
4X

Supplemental Figure 1. Detection of *Prlhr*- and *Npffr2*-expressing neurons in projection targets of NTS *Prlh* neurons. Shown are images of GFP-containing neurons (green) in $Prlhr^{Cre}$- and $Npffr2^{Cre}$-reporter animals (representative of n=2 animals per line). Shown are the DVC and the noted major projection targets of NTS *Prlh* neurons. Representative images showing the distribution of Prlhr (left) and Npffr2 (right) expression in the **DVC** (A,B), **PBN** (C,D), hypothalamus (**HYPO**) (E,F), **PVT** (G,H), **PVH** (I,J), **BNST** (K,L). All images were taken at same magnification except hypothalamus (E,F) and BNST (K,L) ; scale bar equals 100 µm. For hypothalamus (E,F) and BNST (K,L), images were taken at the same magnification; scale bar equals 200um.

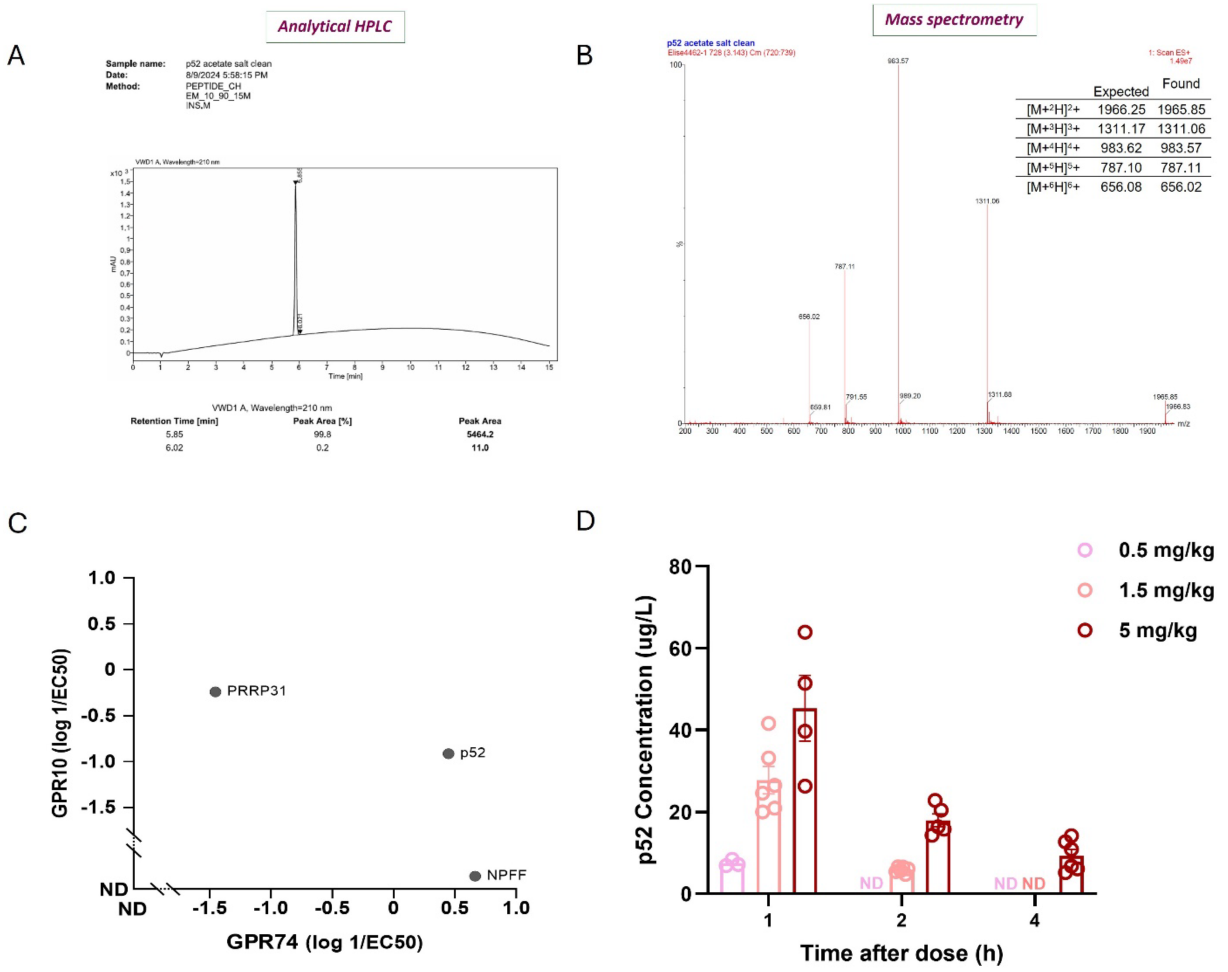

Supplemental Figure 2. Quality analysis, *in vitro* potency, and clearance from the blood of mice for p52. A Analytical RP-HPLC spectrum for p52 was recorded using an Agilent 1260 Infinity system. **B** Spectrographic data from the analysis of purified p52 by single quadrupolar LC/MS using a Waters Mass Lynx 3100 platform. **C** Peptide *in vitro* potencies determined in CHO cells transfected with mouse GPR10 or GPR74 receptor. For GPR74 potency was determined for peptide mediated inhibition of forskolin stimulated cAMP accumulation, as described (48). For GPR10 potency was determined in calcium mobilization assay in cells incubated with FLuo-4AM calcium assay kit and measured on a FLIPR TETRA (Molecular Devices, Sunnyvale, CA). Shown are log(1/EC50) (1/nM) values for cAMP generation via GRP10 and GPR74 for p52, PrRP-31, and NPFF. ND- not detected. **D** Mice were injected with the indicated doses of p52. Blood was drawn at the indicated times after dosing and p52 was quantified by LC-MS. Shown are mean +/- STD; individual measurements are shown, as well; n=6/group. ND- not detected.

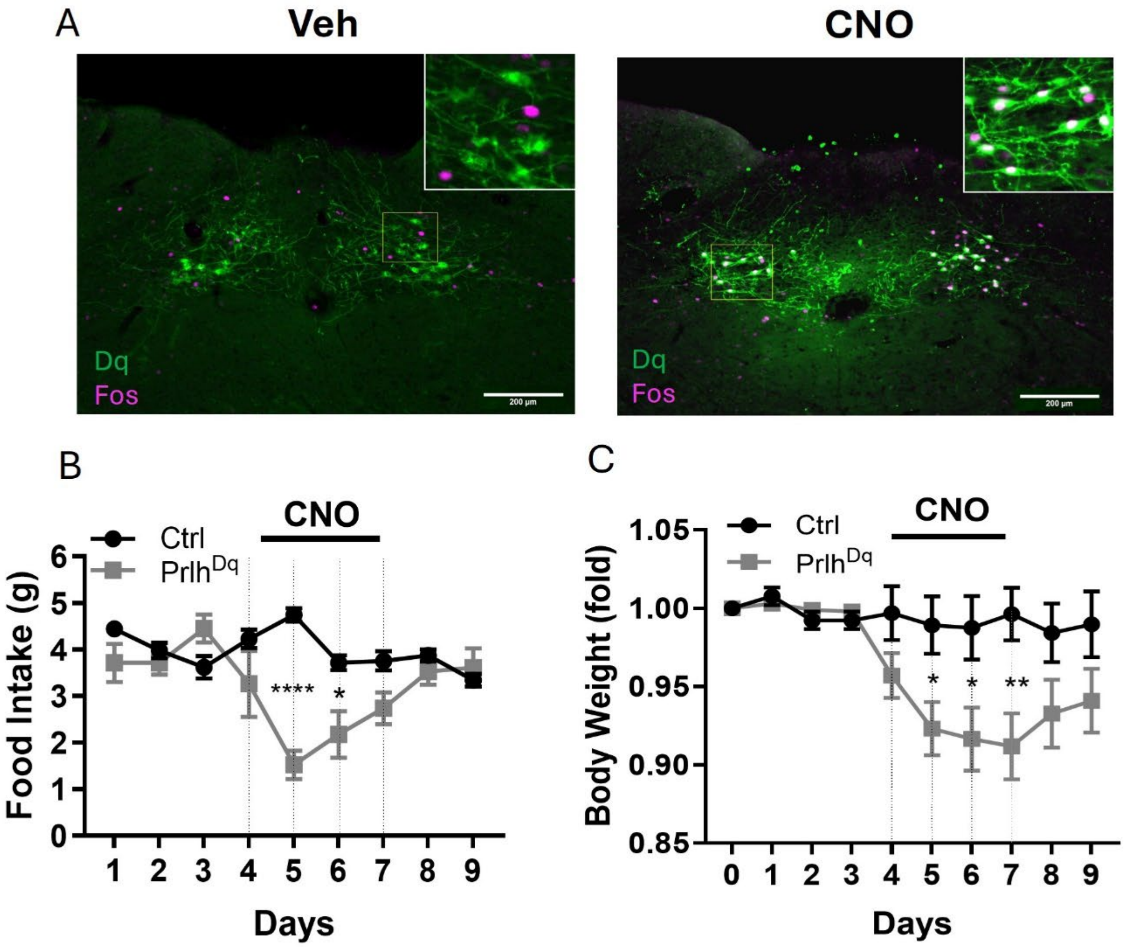

Supplemental Figure 3. Chemogenetic activation of NTS^{Prlh} neurons decreases food intake and body weight. A Representative image showing mCherry-IR (Dq, green (pseudo colored)) and Fos-IR (magenta) in the $NTS^{Prlh-Dq}$ mice following treatment with saline (left, Veh) or CNO (right, 1mg/kg, IP) for 2 h before perfusion. **B, C** Food intake (B) and body weight (C) in control (n=7; black line) and $NTS^{Prlh-Dq}$ (n=8; grey line) mice treated with saline or CNO (1mg/kg, IP, twice daily). All graphs: Shown is mean ± SEM. Two-way ANOVA, Šídák's multiple comparisons test was used; All images were taken at same magnification; scale bar equals 200 µm. p values are shown for significant comparisons. *: p<0.05, **: p<0.01, ***p<0.001, ****: p<0.0001.
